# Biomechanical Effects of Different Load Cases with an Implant-Supported Full Bridge on Four Implants in an Edentulous Mandible: A Three-Dimensional Finite Element Analysis (3D-FEA)

**DOI:** 10.3390/dj11110261

**Published:** 2023-11-07

**Authors:** Árpád László Szabó, Danica Matusovits, Haydar Slyteen, Éva Ilona Lakatos, Zoltán Baráth

**Affiliations:** 1 Department of Prosthodontics, Faculty of Dentistry, University of Szeged, Tisza Lajos krt. 64-66., 6720 Szeged, Hungary; szabo.arpad@stoma.szote.u-szeged.hu (Á.L.S.); matusovits.danica@stoma.u-szeged.hu (D.M.); 2 Department of Structural Mechanics, Faculty of Civil Engineering, University of Technology and Economics, Budapest, Műegyetem rkp. 3., 1111 Budapest, Hungary; haydar.slyteen@edu.bme.hu (H.S.); lakatos.eva@emk.bme.hu (É.I.L.)

**Keywords:** dental implants, dental stress analysis, dental occlusion, finite element analysis, implant-supported, edentulism

## Abstract

The long-term success and predictability of implant-supported restorations largely depends on the biomechanical forces (stresses) acting on implants and the surrounding alveolar bone in the mandible. The aim of our study was to investigate the biomechanical behavior of an edentulous mandible with an implant-supported full bridge on four implants under simulated masticatory forces, in the context of different loading schemes, using a three-dimensional finite element analysis (3D-FEA). A patient-specific 3D finite element model was constructed using pre- and post-implantation computer tomography (CT) images of a patient undergoing implant treatment. Simplified masticatory forces set at 300 N were exerted vertically on the denture in four different simulated load cases (LC1–LC4). Two sets of simulations for different implants and denture materials (S1: titanium and titanium; S2: titanium and cobalt-chromium, respectively) were made. Stress outputs were taken as maximum (P_max_) and minimum principal stress (P_min_) and equivalent stress (P_eqv_) values. The highest peak P_max_ values were observed for LC2 (where the modelled masticatory force excluded the cantilevers of the denture extending behind the terminal implants), both regarding the cortical bone (S1 P_max_: 89.57 MPa, S2 P_max_: 102.98 MPa) and trabecular bone (S1 P_max_: 3.03 MPa, S2 P_max_: 2.62 MPa). Overall, LC1—where masticatory forces covered the entire mesio−distal surface of the denture, including the cantilever—was the most advantageous. Peak P_max_ values in the cortical bone and the trabecular bone were 14.97–15.87% and 87.96–94.54% higher in the case of S2, respectively. To ensure the long-term maintenance and longevity of treatment for implant-supported restorations in the mandible, efforts to establish the stresses of the surrounding bone in the physiological range, with the most even stress distribution possible, have paramount importance.

## 1. Introduction

Partial or complete edentulism—a serious public health issue, affecting an increasing number of number of patients worldwide—is a definite condition, which may be a result of untreated caries and periodontal disease over a long period of time [1]. The use of implant-supported, fixed full-arch restorations is an established treatment alternative for the oral rehabilitation of edentulous patients [2,3]. Implants serve as a framework to transfer functional and parafunctional forces generated during mastication into the peri-implant tissues, and to support the placement of a restoration in either an immediate-loading (IL) or delayed-loading concept (DL) [4]. However, due to the anatomical constraints of the edentulous mandible, or if the quality and the amount of residual alveolar bone is limited, implant-supported prosthetic treatment is impossible without complex surgical interventions preceding implant placement [5,6]. Alveolar crest augmentation, bone grafting, nerve transposition and soft tissue management in the posterior mandible all carry the risk of complications (e.g., loss of soft tissue volume and contours, graft failure, infections), increased morbidity, and poor patient performance [7,8]. Thus, most patients prefer a less invasive and a more economical approach to their dental rehabilitation with shorter recovery time intervals [9]. One of the proposed alternative solutions to these surgical procedures include the use of short and extra-short implants, in which case, increasing the implant diameter, and careful selection of the surgical protocol (one-stage vs. two-stage technique), corresponds to favorable clinical outcomes [10].

The “All-on-Four” (Ao4) treatment concept—devised by Maló et al. (Nobel Biocare, Göteborg, Sweden) in 2003—is another method that allows clinicians to overcome the anatomical limitations of the mandibular bone without necessitating advanced and risky surgical techniques [11,12]. This current strategy for oral rehabilitation involves the placement of four implants in the interforaminal area of the mandible and the premaxillary region—two axial implants, which are positioned in the anterior alveolar region, while the other two implants are tilted (15–45°) in the posterior region—to support IL, one-piece full-arch fixed restorations [13]. With implant angulation in the posterior region, violation of the mandibular nerve is bypassed, the use of longer implants (i.e., resulting in a longer bone-to-implant contact area) is permitted, and the length of the denture cantilevers may also be reduced [14]. Ao4 procedures often include the use of computer-assisted procedures and implant guides, enhancing the safety and reliability of the procedure [15]. Implant survival rate and the short-term success of the Ao4 concept for the rehabilitation of both maxillary and mandibular arches has been reported by numerous short and medium-term studies. The study by Pera et al. compared the clinical outcomes of IL and DL procedures in edentulous maxillae with full-arch fixed prostheses, where all prostheses provided satisfactory function and no significant differences were shown in the cumulative survival rate of implants (IL: 93.3% vs. DL: 94.9%), while mean bone loss was significantly lower in the IL group [16]. In a prospective 6-year study from the same authors, the clinical reliability of the IL protocol was further demonstrated, noting no significant differences in bone loss when comparing tilted vs. axial implants [17]. The 10-year longitudinal study by Maló et al. has demonstrated a 98.2% survival rate of mandibular implants, while a literature summary by Durkan et al. reported success rates ranging between 92.2 and 100%; however, long-term studies with high evidence rigor are lacking [18,19,20].

The long-term success and predictability of implant-supported restorations largely depends on the biomechanical forces (stresses) acting on the implant and the surrounding alveolar bone in the mandible [21]. Load transmission at the bone–implant interface is influenced by a variety of factors, including the length, diameter, form, and surface of the implants; material properties of the implant and/or prosthesis; geometry, quality, and quantity of the residual alveolar bone [22,23,24]; still, the properties of the implants are among the few modifiable biomechanical factors [25]. With the use of a lower number of (tilted) implants, one of the disadvantages of the Ao4 concept is that higher stress and strain around the implant and in the bone may exceed the load bearing capacity of the bone (i.e., overload), resulting in microdamage accumulation and marginal bone resorption [26]. This may threaten primary stability and osseointegration, leading to excessive micromotion and—in severe cases—implant loss/failure [27]. With this in mind, clinicians should be aware of the tensile, compressive, and shear stresses arising in bone from masticatory forces and implants during treatment planning.

Previously, the determination of stress levels in implant and the bones was largely achieved by laboratory measurements performed on cadavers and bone ribbons; however, many of these methods are cumbersome to use [28]. On the other hand, finite element analyses (FEA) to generate qualitative and quantitative biomechanical data in the medical dentistry and field have received substantial attention: they are effective as numerical and/or three-dimensional (3D) methods to assess load distribution and stresses on the restorations, implants, and peri-implant tissues under functional forces [29]. Advantages of FEA in dentistry include their capability to be used for high-throughput analysis and the handling/mimicking complex structures showing irregular geometry (e.g., maxilla and mandible, implants) [30]. However, considerable gaps still exist in the knowledge regarding the biomechanical stresses observed in the peri-implant bone, implants, and prostheses during the treatment of the mandible. For example, while different load cases result in differential stress and strain distributions in the implant and the peri-implant bone, there is no consensus on the type of loading to be favored. Therefore, the aim of the present study was to investigate the biomechanical behavior of an edentulous mandible with an implant-supported full bridge on four implants under simulated masticatory forces in the context of different loading schemes, in a patient-specific finite element model, using a 3D-FEA. The initial hypotheses our study were the following: (i) the occlusion setting (i.e., load case) where the masticatory force covers the entire surface of the denture (including the cantilever) is the most advantageous, when considering stress distributions; (ii) material properties assigned to the denture body and the implant considerably affect stress levels and stress distribution characteristics.

## 2. Materials and Methods

### 2.1. Study Design, Patient Treatment Characteristics

**Figure 1:**
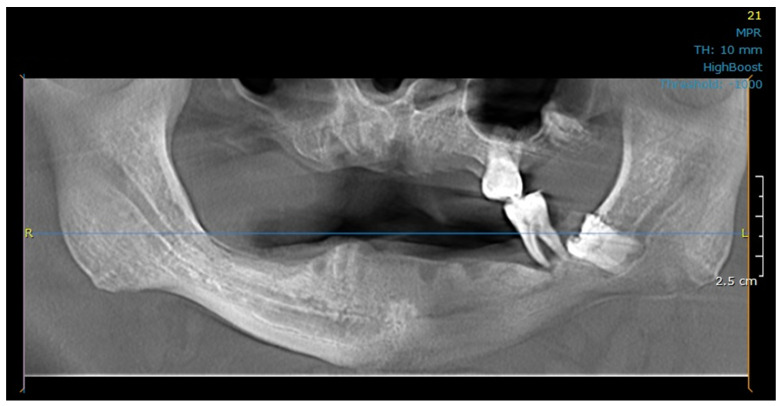
Cone-beam CT (CBCT) image corresponding to the patient’s baseline state.

**Figure 2:**
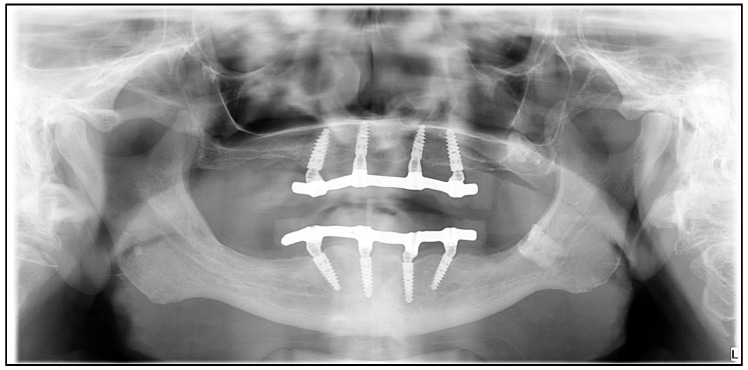
Panoramic radiograph of the patient 4 years post-implant placement.

To perform FEA, a patient-specific finite element model was constructed using pre- and post-implantation computer tomography (CT) images of a 63-year-old male patient with adequate bone supply, who was eligible for treatment with an implant-supported full bridge on four implants. Implant placement occurred 6 months post-extraction. The patient’s final prosthesis consisted of a milled cobalt−chromium (Co−Cr) alloy frame with a cold-curing pour-type acrylic denture base (Vertex Dental B.V., Soesterberg, The Netherlands) and Ivoclar Vivadent (Schaan, Liechtenstein) denture teeth. The cone-beam CT (CBCT) image corresponding to the patient’s baseline state and the panoramic radiograph 4-year post-implant placement are presented in Figure 1 and Figure 2, respectively.

To ensure the most accurate bone modelling possible, finite element models of the trabecular and the compact bone were created by the segmentation of the CT images of the pre-implantation edentulous mandible. This prevented the adverse effect of X-ray image artifacts in the environment of metallic materials on the subsequent material properties. The geometry and precise location of the implants in the jawbone were obtained by processing the post-implantation CT images. The two datasets—obtained by separate segmentations—were fused to create the final model including trabecular bone, compact bone, and implants.

### 2.2. Ethical Considerations

The study was conducted in accordance with the Declaration of Helsinki and national and institutional ethical standards. Ethical approval for the study protocol was obtained from the Human Institutional and Regional Biomedical Research Ethics Committee, University of Szeged (registration number: 158/2021-SZTE [5035]). Written informed consent was obtained from the patient involved in this study.

### 2.3. Modeling

CT images (in dicom format) were imported into the 3D Slicer Computer Aided Design (CAD) software. To find the best display of the mandible, brightness and contrast were adjusted manually on the CT images, which were then loaded into 3D Slicer to create a 3D model by combining the slices together [31]. Segmentation of the mandibular bone was completed manually using the “Threshold” command of the 3D Slicer Segmentation module. After the generation of the initial 3D model, the “Scissor” and “Island” tools were used to eliminate noise (i.e., excessive bone, small islands) from the model. The “Smoothing” command with the median smoothing option—which removed small extrusions and filled small gaps, while keeping smooth contours mostly unchanged—was used to eliminate roughness on the surface of the 3D model [31]. The segmentation of the mandible was finalized by the elimination of the residual metal support by the “Scissor” tool. To generate the cortical (compact) bone section, the “Hollow” command was used to create a new segment—which was a replica of the mandibular surface—at a thickness of 2.5 mm, with the assumption that cortical bone layer thickness was regular. The trabecular (cancellous) bone section was simulated by subtracting the cortical bone segment from the mandible.

The 3D geometry of the cylindrical implants was constructed using the same patient’s CT images, who received four implants in both the maxilla and the mandible, according to the SmartGuide^®^ protocol (iRES^®^, Mendrisio, Switzerland) [32]. The two anterior implants (MultiNeO^TM^ with a Conical Standard (CS) implant-abutment connection platform, Alpha-Bio Tec Ltd., Petah Tikva, Israel) were threaded, with angled multi-unit abutments (Alpha-Bio Tec Ltd., Petah Tikva, Israel; 17°/2.5 mm and 30°/2.5 mm, respectively) and dimensions of 4.2 × 11.5 mm and 3.75 × 13 mm (diameter and length), respectively, were placed straight and parallel to each other. Two distally tilted implants (MultiNeO^TM^ CS, Alpha-Bio Tec Ltd., Petah Tikva, Israel; the implants were threaded with a diameter and length of 4.2 × 11.5 mm) with angled multi-unit abutments (Alpha-Bio Tec Ltd., Petah Tikva, Israel; 17°/2.5 mm) were placed in the posterior region of the mandible [33]. The distance between the anterior two implants in the mandible was 15.5 mm, while the distance between the anterior and posterior implants was 11.0 mm and 9.16 mm, respectively. Steps to generate the 3D view of the implants were similar to those described for the mandible, their positioning inside the mandible was identical to the source material. The resulting CAD models were recorded in “step” and “iges” formats, which could be imported into the ANSYS SpaceClaim software (ANSYS 19.1, Canonsburg, PA, USA) to create the implant and mandible components’ solid body mesh.

### 2.4. Meshing, Boundary Conditions

**Figure 3:**
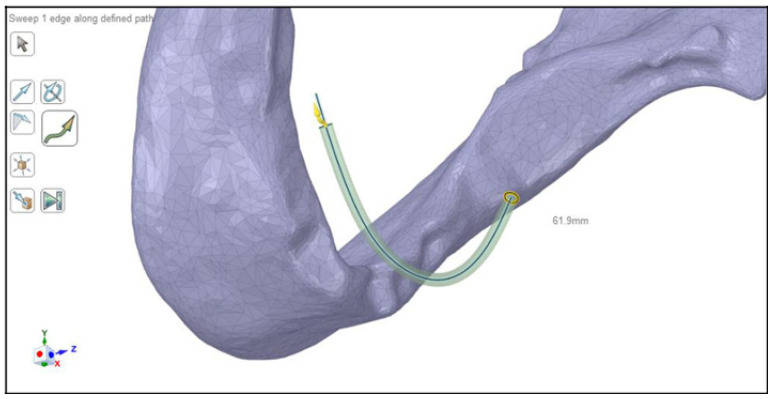
Creation of the simplified denture during the modeling process.

**Figure 4:**
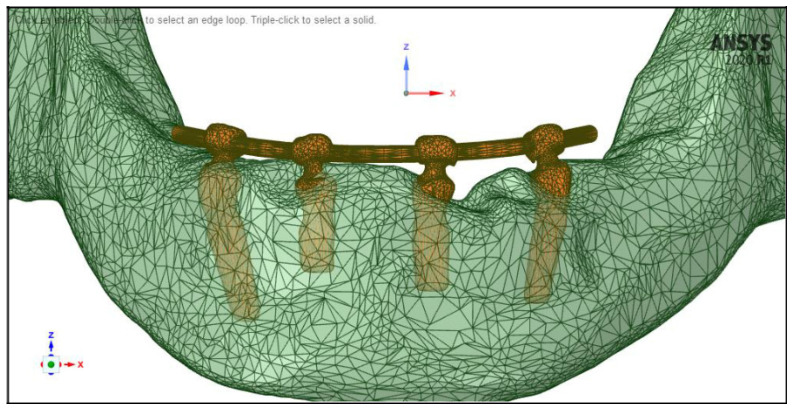
Finite element mesh of the implant-denture and the mandible models.

SOLID187 (a 10-node, higher order 3D element with quadratic displacement behavior, ideal for modeling irregular meshes) and CONTA174 (an 8-node 3D element used to model contact and sliding between surfaces) elements were used to generate the mesh of the mandible and the implants using ANSYS SpaceClaim [34,35]. Element size was adjusted to be finer at the implants and the contact surfaces with the mandible bone, on the other hand, the mesh was coarser at the rest of the mandible body. The number of elements and nodes of the models were 569,588 and 1,430,889, respectively. A simplified denture was included in the simulations with a metallic base and applied with a realistic geometry. The denture was assumed to be a horseshoe-shaped curved cylinder, with a diameter of 2 mm, running about 2 mm over the mandible surface, which was created by the “Spline” (used to create a curved line), “Pull” (used to generate volume elements from surface elements, or surface elements from line elements), and “Fill” (used to convert the object into a solid body) commands of ANSYS SpaceClaim (Figure 3). After checking for vertical alignment with the implants, the denture was integrated into the implant mesh, creating a single facet interpenetrating the mandible, which was then subtracted from the model of the cortical and trabecular bone. Following the automatic and manual geometric repair of meshing errors, the facet mesh was converted into solid body mesh (Figure 4). As the present study focused on the functional behavior of the implants inside of the mandibular bone, the boundary conditions were fixed, the movements of the temporo-mandibular joint were neglected, and a fixed support was applied close to the vicinity of the joint [36].

In order to keep the number of elements at a reasonable level, the model considers the wire supporting the prosthesis, instead of the entire prosthesis, as the medium transmitting the masticatory loads to the implant. As the stiffness of the entire prosthesis is mainly provided by the Ti wire mentioned above, in our analyses, the distribution of the masticatory forces on the denture and the wire are assumed to be similar. For similar reasons, to reduce the complexity of the model, the geometry of the implants obtained from the post-implantation CT image of the same patient was used.

### 2.5. Material Properties

The peri-implant bone in the model was made up of cortical and trabecular bones with a transition region that extends past the implant’s outermost edge. The interface between the bone and the implant was set as bonded; osseointegration was assumed to be 100% [37]. Based on previous literature findings, the material properties, which define the physical properties of the modelled structures, were entered into the software, according to the values presented in Table 1. The physical features of the peri-implant bone were modelled to reflect the features of type II bone, according to the Lekholm and Zarb classification [38]. All parts in the model were accepted as homogeneous, isotropic, and linear elastic [39]. Two sets of simulations were carried out to simulate framework material changes: in the first set of simulations (denoted as S1), the denture body and the implant bodies were assigned the same material (titanium, i.e., TiAl6V4), while in the second set of simulations (denoted as S2), different material properties were assigned to the implant bodies (TiAl6V4) and the denture bodies (a cobalt−chromium alloy 70–30%, i.e., CO−CR-01-P.30CR).

### 2.6. Loading, Occlusal Cases

**Figure 5:**
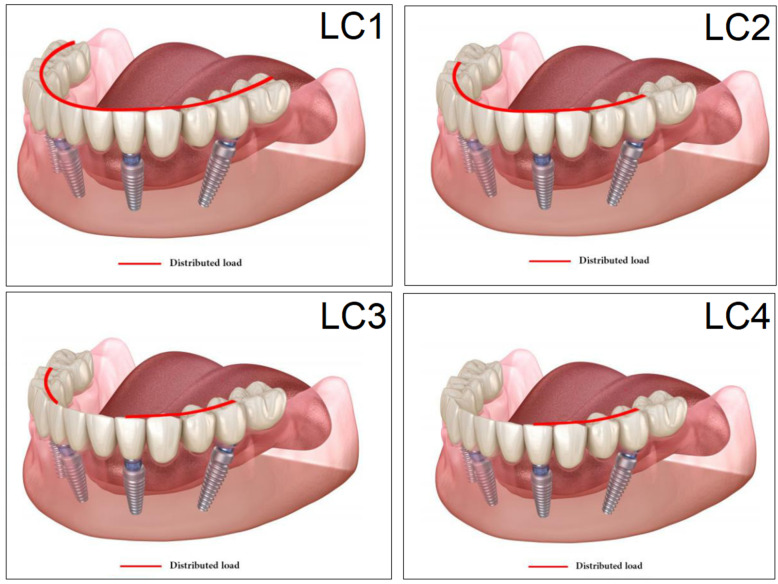
Load cases (LC1–4) used in the study. The red line represents the distributed load applied in the finite element analyses (FEA).

The finite-element simulation to model the state of the peri-implant bone and the stress distribution was carried out using the ANSYS Workbench software (ANSYS 2020 R1, Canonsburg, PA, USA). The aim of the present study was to investigate the effect of occlusion settings, i.e., the appropriate location of the masticatory force, and therefore, for the sake of comparability, the vertical components of the masticatory forces were included in the calculations; these were set at 300 N to be exerted on the denture in four different simulated load cases [44], as seen in Figure 5 and described below.

**Load case 1 (LC1):** the distributed masticatory force that covers the entire surface of the denture.

**Load case 2 (LC2):** similar to LC1, but the force excludes the cantilevers of the denture stretching behind the terminal implants.

**Load case 3 (LC3):** the masticatory force was exerted on the denture at the premolar region, at the area extending between the front and side implants.

**Load case 4 (LC4):** similar to LC3, a nonsymmetrical distributed force, but applied on only one side of the denture.

In the case of linear analysis, it is assumed that the relationship between the force acting on the examined body and the deformation caused by the mentioned force is linear [45]. In our analyses, positive values (+) represent tension, while negative values (−) represent compression stress. Stress outputs for the mandible from the ANSYS Workbench were taken as maximum principal stress (or first principal stress, [P_max_], representing the strongest tensile stress at the point of interest), minimum principal stress (or third principal stress, [P_min_], representing the strongest compressive stress), and equivalent stress (or von Mises stress, [P_eqv_], representing the stress around the implant, i.e., where the load is transferred to the bones).

### 2.7. Statistical Analysis

The results of FEA do not have variance, therefore there was no need to perform statistical analysis.

## 3. Results

**Figure 6:**
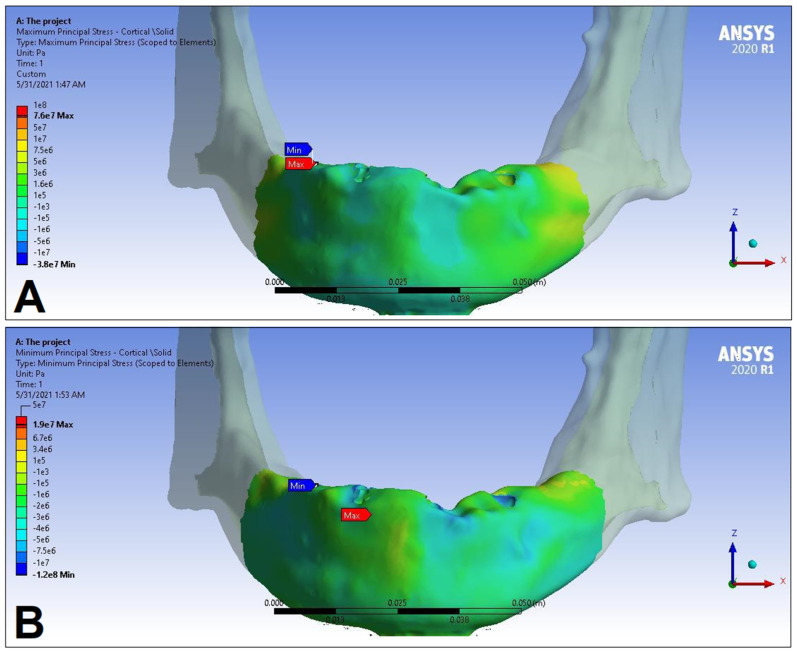
Maximum (P_max_, **A**) and minimum (P_min_, **B**) principal stress distributions in the cortical bone segment of the mandible for the S1 LC1 case. The heatmap shows the distribution of stresses according to the color scale, while the maximum and minimum values for stresses are also denoted (e.g., 8E3 corresponds to 8 × 10³).

**Figure 7:**
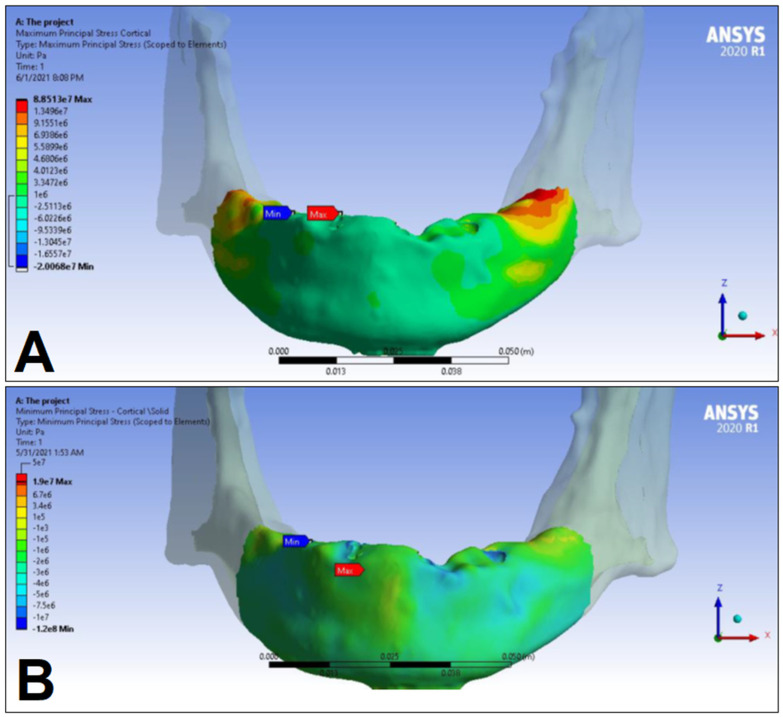
Maximum (P_max_, **A**) and minimum (P_min_, **B**) principal stress distributions in the cortical bone segment of the mandible for the S2 LC1 case. The heatmap shows the distribution of stresses according to the color scale, while the maximum and minimum values for stresses are also denoted (e.g., 8E3 corresponds to 8 × 10³).

**Figure 8:**
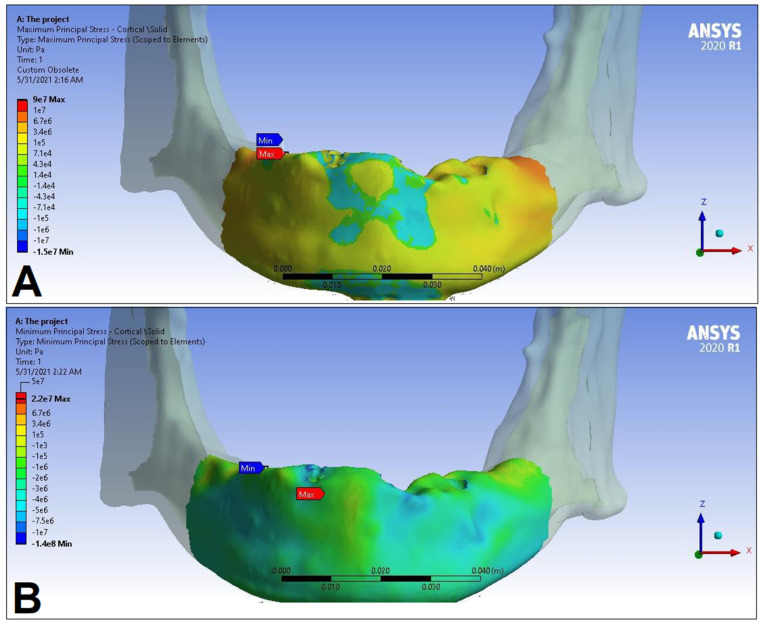
Maximum (P_max_, **A**) and minimum (P_min_, **B**) principal stress distributions in the cortical bone segment of the mandible for the S1 LC2 case. The heatmap shows the distribution of stresses according to the color scale, while the maximum and minimum values for stresses are also denoted (e.g., 8E3 corresponds to 8 × 10³).

**Figure 9:**
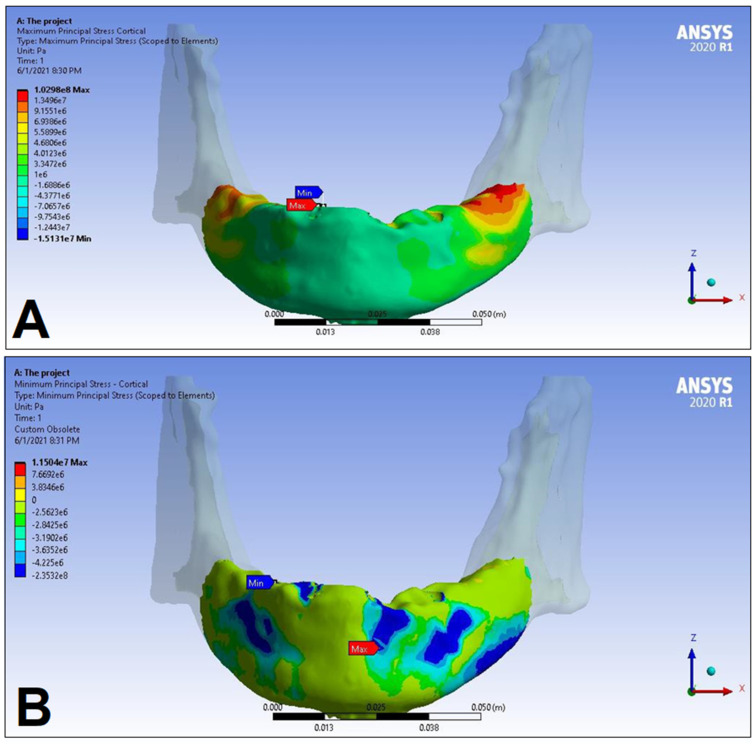
Maximum (P_max_, **A**) and minimum (P_min_, **B**) principal stress distributions in the cortical bone segment of the mandible for the S2 LC2 case. The heatmap shows the distribution of stresses according to the color scale, while the maximum and minimum values for stresses are also denoted (e.g., 8E3 corresponds to 8 × 10³).

**Figure 10:**
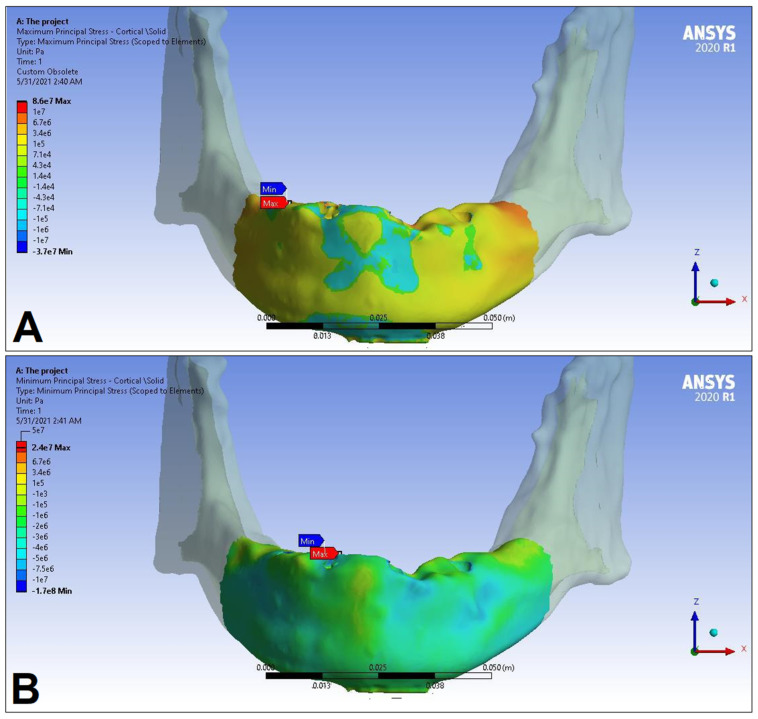
Maximum (P_max_, **A**) and minimum (P_min_, **B**) principal stress distributions in the cortical bone segment of the mandible for the S1 LC3 case. The heatmap shows the distribution of stresses according to the color scale, while the maximum and minimum values for stresses are also denoted (e.g., 8E3 corresponds to 8 × 10³).

**Figure 11:**
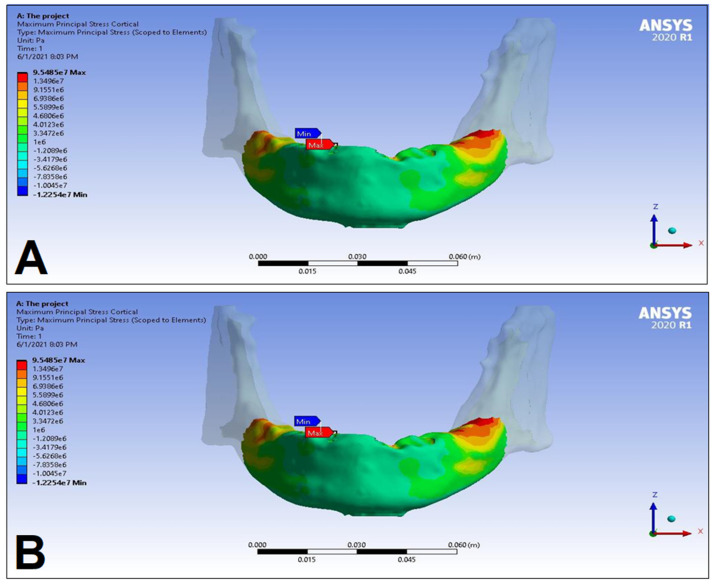
Maximum (P_max_, **A**) and minimum (P_min_, **B**) principal stress distributions in the cortical bone segment of the mandible for the S2 LC3 case. The heatmap shows the distribution of stresses according to the color scale, while the maximum and minimum values for stresses are also denoted (e.g., 8E3 corresponds to 8 × 10³).

**Figure 12:**
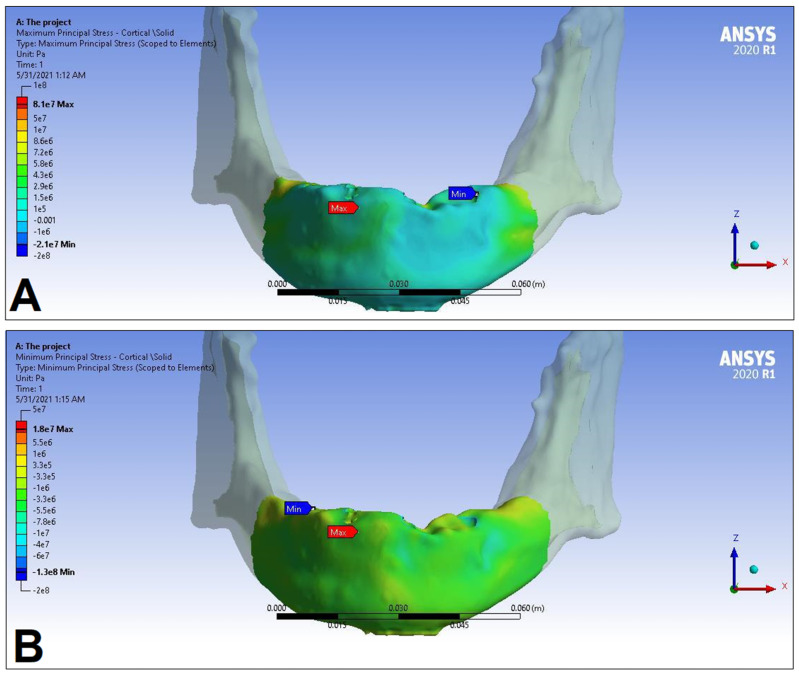
Maximum (P_max_, **A**) and minimum (P_min_, **B**) principal stress distributions in the cortical bone segment of the mandible for the S1 LC4 case. The heatmap shows the distribution of stresses according to the color scale, while the maximum and minimum values for stresses are also denoted (e.g., 8E3 corresponds to 8 × 10³).

**Figure 13:**
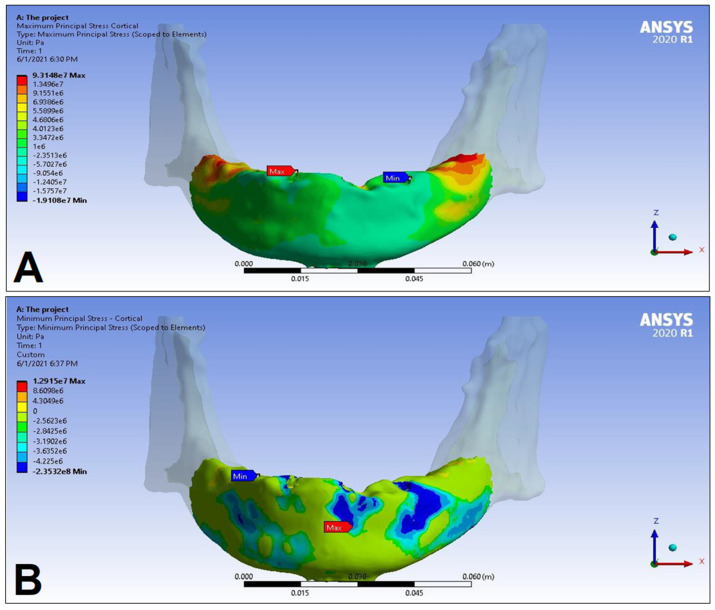
Maximum (P_max_, **A**) and minimum (P_min_, **B**) principal stress distributions in the cortical bone segment of the mandible for S2 LC4 case. The heatmap shows the distribution of stresses according to the color scale, while the maximum and minimum values for stresses are also denoted (e.g., 8E3 corresponds to 8 × 10³).

In the following, the simulation results of the four sets of masticatory load cases (LC1–4), corresponding to different implant-denture material configurations (S1 and S2) are presented, expressed as the maximum principal stress (P_max_), minimum principal stress (P_min_), and equivalent stress (P_eqv_) values in the cortical and the trabecular bone (Table 2). Additionally, stress maps and range scales (shown in different colors) for maximum and minimum principal stresses are demonstrated for each load case for the cortical (Figure 6, Figure 7, Figure 8, Figure 9, Figure 10, Figure 11, Figure 12 and Figure 13) and trabecular bodies (Supplementary Material S1), respectively. For more visibility, implants were not included in these stress maps.

Overall, based on the stress maps for principal stress distribution, the highest stress values were always seen at the implant—bone interface. Compressive stress values were 1.5–2.5-times higher and 1.1–1.4-times higher than tensile stress values in the cortical bone and trabecular bone, respectively (Table 2). The highest maximum principal stress values were observed for the load case LC2, both regarding the cortical bone (S1 P_max_: 89.57 MPa, S2 P_max_: 102.98 MPa) and the trabecular bone (S1 P_max_: 3.03 MPa, S2 P_max_: 2.62 MPa). The highest tensile stress for LC2 was seen near the top of the third implant for the cortical bone, and near the top of the second implant for the trabecular bone. The highest minimum principal stress values for the cortical bone were seen in the S2 LC2 (P_min_: −265.35 MPa) and S1 LC3 cases (P_min_: −172.30 MPa), while in the case of the trabecular bone, these were seen in the case of LC4 (S1 P_min_: −3.49 MPa, S2 P_min_: −3.52 MPa), respectively, which were seen near the top of the second implant. Nevertheless, all other load cases (including LC3 and LC4) showed higher P_max_ and P_min_ values for both simulations and bone segments, compared to LC1, where the force covers the entire surface of the denture, including the extension surface (cortical bone: S1 P_max_: 76.39 MPa, S2 P_max_: 88.51 MPa; S1 P_min_: −115.30 MPa, S2 P_min_: −222.76 MPa; trabecular bone: S1 P_max_: 2.49 MPa, S2 P_max_: 2.24 MPa; S1 P_min_: −2.81 MPa, S2 P_min_: −2.89 MPa). Peak equivalent stress values were highest in the case of LC1 (166.40 MPa) and LC2 (279.69 MPa) for S1 and S2, respectively; the lowest equivalent stress was observed at LC4 (142.27 MPa) for S1, and LC1 (244.92 MPa) for S2.

Peak maximum principal stress values in the cortical bone were 15.87%, 14.97%, 11.50%, and 14.97% higher in the case of S2, for the LC1, LC2, LC3, and LC4 load cases, respectively. In light of this, peak minimum principal stress values in the cortical bone were 93.20%, 94.54%, 46.61%, and 87.96% higher in the case of S2, for the LC1, LC2, LC3, and LC4 load cases, respectively. Peak maximum principal stress values in the trabecular bone were 11.16%, 15.65%, 15.87%, and 15.87% higher in the case of S1, for the LC1, LC2, LC3, and LC4 load cases, respectively. On the other hand, differences in the peak minimum principal stress values in the trabecular bone were considerably smaller, i.e., 2.85%, 1.20%, 0.0%, and 0.86% higher in the case of S2, for the LC1, LC2, LC3, and LC4 load cases, respectively. Equivalent (von Mises) stress values were higher 47.19%, 68.12%, 61.58%, and 83.29% higher in the case of S2, for the LC1, LC2, LC3, and LC4 load cases, respectively (Table 2).

## 4. Discussion

This study herein evaluated the biomechanical effects of different occlusion/load cases and implant-denture material properties in an edentulous mandible (constructed using authentic CT scans of a patient) with an implant-supported full bridge on four implants. Due to the bone’s elastic material properties, tensile and compressive stress values were deemed appropriate to evaluate biomechanical properties in this study [46]. Based on the results of the analyses, the LC1 modeled—where masticatory forces covered the entire surface of the denture, including the cantilever—was noted as the safest option, confirming our initial hypotheses. This load case was characterized by the most uniform stress distribution, and the lowest peak maximum principal stress and minimum principal stress values in the mandible body, throughout all simulations. On the other hand, LC2—the load case where the force excluded the cantilevers of the denture extending behind the terminal implants—showed the highest peak tensile stress values in both cortical and trabecular bone for S1 and S2, respectively; therefore, it was the least desirable option in our analyses. For compressive stress, the situation was a bit more complex: in case of the cortical bone, OC2 for S2 and OC3 for S1 showed the highest values (−265.35 MPa and 172.30 MPa, respectively), while in the case of the trabecular bone, OC4 had the highest peak values (both for S1 and S2). Overall, all other load cases in most simulation parameters showed higher stress values than OC1. As seen on the stress distribution maps, noted stress values were peak values denoted at a specific position, however, in reality, these maximum stresses occur as a load transmitted at the bone–implant interface, not at a single point [47]. Although comparisons may be hindered by the different model characteristics set by researchers, our results have yielded similar tensile and compressive values in the same order to other previously published reports assessing stresses on mandibular bone tissue [4,21,36,37,46,48,49,50]. The mandibular bone adapts to its loading and responds to stresses by bone formation or resorption, i.e., neither unloaded nor overloaded areas are desirable due to long-term consequences [51]. Thus, the longevity of an implant may be ensured by keeping the stresses of the bone in the physiological range, with the most even stress distribution possible [52]. Overloading and subsequent bone resorption would occur if the tensile and compressive values exceed the physiological limits posed by the ultimate strength of the bone; stress values resulting from our FEA were below these physiological limits in all simulations and load cases [53]. In addition, the rigid full-arch restoration and the spread of the implants in the mandible will further reduce stress on an individual implant-level.

Among the implant-supported full bridge restorations on four implants, the Ao4 treatment concept has been widely popularized in the recent years for the oral rehabilitation of an atrophic mandible, due to high level of functionality and patient satisfaction rates [54]; the clear advantages of this technique include the small number of implants needed, less complex surgery, the use of longer, tilted distal implants (resulting in a shorter cantilever), and large inter-implant distances, leading to improved anchorage to the bone and higher primary stability [55]. Primary stability—especially in the case of immediately loaded implants—is one of the most critical factors influencing successful implant placement [56]. To ensure successful osseointegration (and to avoid non-mineralized, fibrous encapsulation) micromotion values should be under 50–150 µm [57]. As the Ao4 concept relies on a lower number of implants in the mandible, the characteristics of individual implants become all the more relevant in treatment planning. Based on various clinical reports, the use of shorter implants has been discouraged, due to their lower success rate; on the other hand (when implant diameter is kept constant), there are considerable benefits to increasing implant length in enhancing bone-implant contact area and primary stability, but only up to a cut-off point of around 12–15 mm [58]. Studies have demonstrated that increased stress in the implant and in the peri-implant area is proportional to longer cantilever lengths. For example, according to the study by Bevilacqua et al., it was shown that decreasing the cantilever length—irrespective of distal implant inclination angles (0°, 15°, 30° and 45°)— led to a reduction in all modelled stress values [59]. Thus, due to the tilted distal implants, shorter cantilever length will subsequently lead to lower stress and strain values. The 3D-FEA study of Liu et al. highlighted this, when assessing the stress distributions of immediate- and delayed-loaded Ao4 implants in an edentulous maxilla; their study included various implant inclination angles (0°, 15°, 30°, and 45°) for posterior implants, where a multivectoral load of 150 N was applied to the distal cantilever of the superstructure. In the immediate-loading cases, the highest P_max_ and P_min_ values in the cortical bone were seen for the 0° inclined implants, while these stresses decreased by 24.91% and 53.00%, respectively, for the 45° implants. The average P_max_ and P_min_ values (corresponding to the load on the entire model) decreased with the increasing inclination angles in all measurements [36]. Malhotra et al. studied the effect of distal implant angulation with different cantilever lengths in a mandibular Ao4 model, where unilateral and bilateral axial and oblique forces were applied and von Mises stress and strain distribution was measured. Their results showed that there were significant differences in the P_eqv_ values between 30° and 45° implants, while no such differences were shown for increasing the cantilever length from 4 mm to 12 mm [60]. Their results are in concordance with the report of van Zyl et al., demonstrating that the ideal level of cantilever loading exists up to 15 mm, while over this threshold value, buccal and lingual cortical plates may be under considerably greater stresses, risking implant failure [61]. Overall, their studies have also underlined that longer cantilever lengths should be avoided. On the other hand, the tilting of distal implants may also lead to increased stress to the peri-implant bones. This has been demonstrated by Almeida et al., showing in their analyses that 45° implants had 32% (under axial load) and 48% (under oblique load) higher P_max_ values and 73% higher P_eqv_ values compared to the vertical (0°) implants in a FEA model of an atrophic maxilla [62].

One of the main findings of the current study is the considerable effect that the load positions had on the distribution of the tested stresses. It should also be noted that in our FEA model, peak stress values were measured near the implant−bone interface, which may be explained by the stress distribution characteristic of the cylindrical implants modeled in the present study [63]. The geometry of the implant body and surface thread may have considerable effects on load transfer characteristics: while smooth, cylindrical implants may transfer dangerous shearing effects at the bone—implant interface (resulting in higher rates of implant failure) and through the introduction of (micro)threads to the implant architecture as a surface function these shear forces may transform into more tolerant force forms transferred to the bone surface [64]. Wu et al. performed an in vitro experiment coupled with 3D-FEA to assess the effects of implant design on the stress distribution in mandibular Ao4 implants; in their study, three distinct loading positions were defined (i.e., at the central incisor area, and at molar regions with or without cantilever load) and they showed that the peak stress values were 36–62% and 45–57% higher, respectively, in the non-cantilever load case [65]. Horita et al. performed a FEA-based micromotion and stress analysis in an edentulous mandible; in their analysis, peak principal compressive stress values were higher in the immediate-loading case, both with and without cantilever loading, while for non-cantilever loading, a ~45–50% reduction in stress values were shown. Their 3D model showed peak principal compressive and tensile stress in the bone around the neck of the right distal implant in the tested cases, which may be due to the relatively high Young’s modulus of the cortical bone, which lies in the closest vicinity of the occlusal loading area and the implant neck. In this report, the framework material did not have a pronounced effect on the results [66].

The advantage of using standardized FEA models to compare the stress distribution of various load cases is that the intended (study) factors may be changed at will while keeping all other study factors constant, ensuring all changes in the simulation outcome are due to the effect of the studied variable [36,39,62]. In contrast to our initial hypotheses, the framework applied (S1 and S2)—based on the FEA results—has a relatively small effect regarding peak maximum principal stress values in the cortical bone (difference: 11.50–14.97%) and trabecular bone (difference: 11.16–15.87%); on the other hand, peak minimum principal stress values in the cortical bone (difference: 46.61–94.54%) and equivalent (von Mises) stress values (difference: 47.19–83.29%) were considerably higher in the case of S2 (i.e., the simulated Ti and Co−Cr framework). The finite element modeling study by Bhering et al. compared biomechanical stresses in the maxilla for Ao4 and “All-on-Six” (Ao6) implants using different framework materials (Ti, Co−Cr, and Zr); the study showed that P_max_, P_min_, and P_eqv_ values for the cortical and cancellous bone and implant displacement were significantly lower for the Ao6 model, associated with the higher number of implants. On the other hand, their results showed that the different framework materials had no considerable effect on implant displacement or on any of the stresses modelled [67]. A finite-element micromotion analysis performed by Siguira et al.—using parallel-implant and Ao4 implant configurations in an edentulous mandible—highlighted the influence of trabecular bone thickness (defined as high and low-density) on preventing micromotion, while cortical bone thickness seemingly played a smaller role. In their Ao4 model, the maximum micromotion for non-cantilever loading was one-third of that with cantilever loading [68].

The present study possesses several limitations that should be acknowledged. To perform our analyses, some biologically complex (e.g., anatomical complexity of the mandible, macrostructure, and microstructure of the implants, boundary conditions) and variable factors were considered constant out of necessity, e.g., all materials were considered homogeneous, isotropic, and linear elastic, a type II bone was used for simulation, and osseointegration was assumed at 100% [36,39,62]. The present study employed a patient-specific 3D finite element model, where the patient had adequate bone supply and was eligible for treatment with an implant-supported full bridge on four implants. Nonetheless, additional studies involving patients with limited bone supply and/or underlying conditions, which would potentially affect short and long-term implant survival, are desperately needed. While it is important to highlight that modeling the size of the implants was accurate and the model was based on CT scans of a patient with adequate bone supply, anisotropy better reflects material properties in dentistry, and osseointegration is a gradual process; thus, changes in these parameters may lead to different results in the FEA. The reliability of the 3D FEA stress analysis largely depends on the number and ratio of elements and nodes (including the use of higher order elements) in the model [32,35,62]; in our case, the number of elements and nodes is in line with other studies already published to ensure maximum sensitivity of the model. Nevertheless, increasing their number would further enhance the reliability of the simulations. As it is well known, mastication is a sophisticated and complex process, which makes its accurate estimation difficult for FEA studies. In our case, masticatory forces, which are multivectoral (vertical, horizontal, and oblique) under real circumstances, were modeled using a linear, continuous force exerted vertically on the simplified denture [69]. Therefore, in future studies, the introduction of multiple-bite forces, load configurations, load directions, magnitudes, material properties, and implant types are needed to complement and confirm our findings. Nevertheless, more robust evidence—such as long-term clinical studies—would be needed to assess the real-life clinical consequence of the presented occlusal cases on implant survival and bone resorption.

## 5. Conclusions

Within the limitations of the study, the following conclusions were drawn.

1. Among our mandibular models, the load case where masticatory forces covered the entire mesio−distal surface of the denture, including the cantilever, was identified as the most advantageous (with the most uniform stress distribution and the lowest peak stress values), while the load case where the modelled masticatory forces excluded the cantilevers was observed as the least desirable option in our analyses

2. The material properties of the denture in our models had a considerable influence on the peak minimum principal stress values in the cortical bone and on equivalent stresses, while the same was not noted in for peak maximum principal stresses

To ensure the long-term maintenance and longevity of an implant-supported full bridge on four implants in the mandible, efforts to establish the stresses of the surrounding bone in the physiological range—with the most even stress distribution possible—have paramount importance. Therefore, additional data and further studies on stress distributions associated with different load cases and in models originating from patients with varying bone supply may provide valuable information to clinicians when making decisions on restorations and occlusal loading conditions.

## Figures and Tables

**Table 1:** Material properties used in FEA.

Materials	Density ρ [g/cm^3^]	Young’s Modulus E [GPa]	Poisson’s Ratio v
**Titanium** [40,41] **(TiAl6V4)**	4.51	102	0.36
**Cobalt−Chromium** [40,41] **(CO−CR-01-P.30CR)**	10	210	0.29
**Cortical bone** [42,43]	1.6	15	0.3
**Trabecular bone** [42,43]	0.2	0.096	0.3

**Table 2:** Peak tension (P_max_), compression (P_min_) stress, and equivalent stress (P_eqv_) values in the different parts of the mandibular bone structure [MPa].

		LC1		LC2		LC3		LC4	
		S1	S2	S1	S2	S1	S2	S1	S2
**Cortical bone**	P_max_ [MPa]	*76.39*	*88.51*	**89.57**	**102.98**	85.63	95.48	81.02	93.15
	P_min_ [MPa]	*−115.30*	*−222.76*	−136.4	**−265.35**	**−172.30**	−252.61	−125.20	−235.32
**Trabecular bone**	P_max_ [MPa]	*2.49*	*2.24*	**3.03**	**2.62**	2.95	2.52	2.92	2.59
	P_min_ [MPa]	*−2.81*	*−2.89*	−3.34	−3.38	−3.25	−3.25	**−3.49**	**−3.52**
P_eqv_ [MPa]		**166.40**	*244.92*	166.36	**279.69**	164.36	265.58	*142.27*	260.77

The values in *italics* represent the lowest, while values in **boldface** represent the highest tension stress (P_max_), compression stress (P_min_), and equivalent stress (P_eqv_) values in each case; **LC1–LC4:** load case 1–4; **S1:** material assigned for denture body and implant bodies is TiAl6V4; **S2**: material assigned for implant bodies was TiAl6V4, while this was a cobalt−chromium alloy for the denture body; **MPa:** megapascal.

## Data Availability

All data generated during the study are presented in this paper and its supplementary materials.

## Supplementary Materials

The following supporting information can be downloaded at: https://www.mdpi.com/article/10.3390/dj11110261/s1, Supplementary Material S1: stress distributions for the trabecular bone segments.
