# Supplementary material for: Biomechanical Effects of Different Load Cases with an Implant-Supported Full Bridge on Four Implants in an Edentulous Mandible: A Three-Dimensional Finite Element Analysis (3D-FEA)
Source: Dent J (Basel). 2023 Nov 7;11(11):261. doi: 10.3390/dj11110261 (PMC10670282; doi:10.3390/dj11110261)
Supplement: Supplementary file 1 [file dentistry-11-00261-s001.zip › dentistry-2512860-supplementary.pdf]

Supplementary material S1

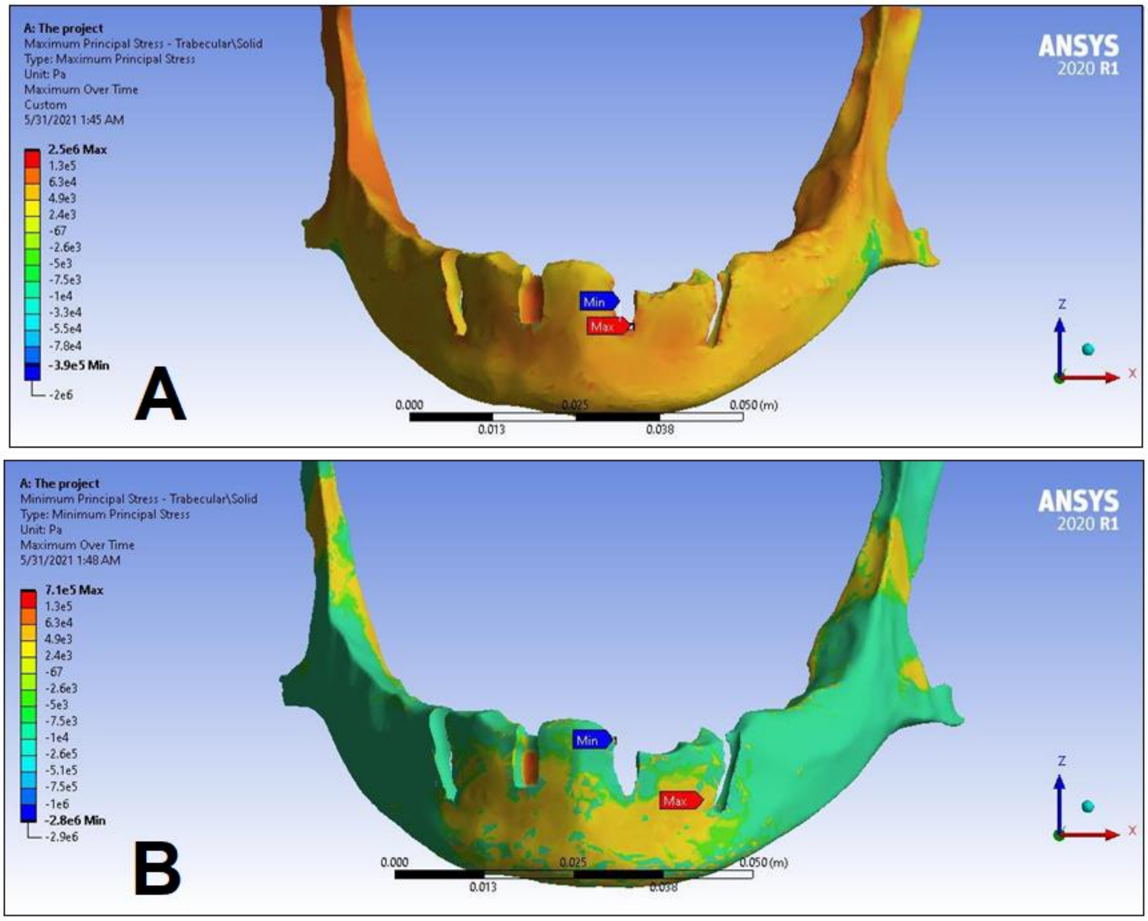

Supplementary figure S1. Maximum (P_{max}, A) and minimum (P_{min}, B) principal stress distributions in the trabecular bone segment of the mandible for the S1 OC1 case. The heatmap shows the distribution of stresses according to the color scale, while the maximum and minimum values for stresses are also denoted.

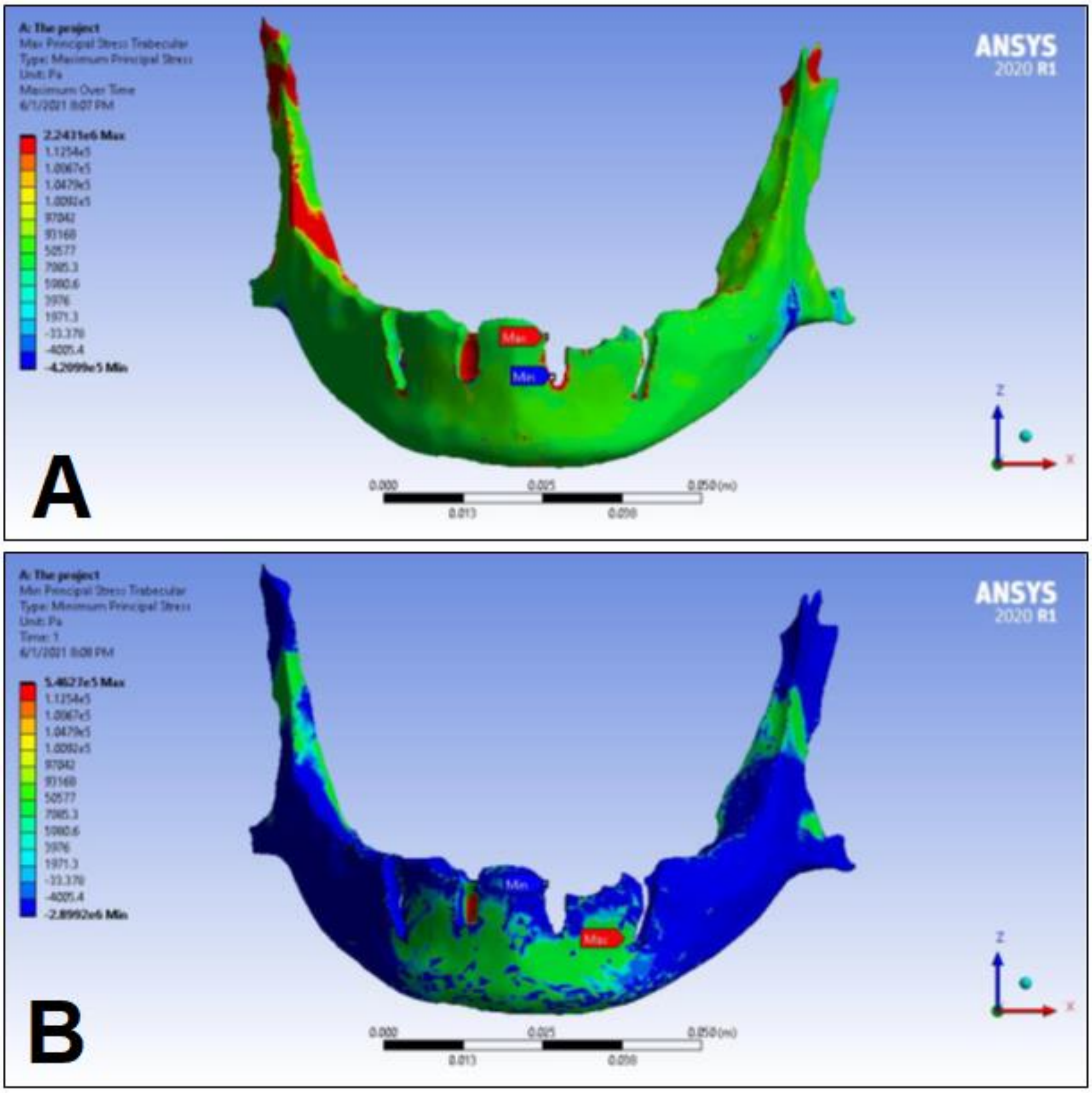

Supplementary figure S2. Maximum (P_{max}, A) and minimum (P_{min}, B) principal stress distributions in the trabecular bone segment of the mandible for the S2 OC1 case. The heatmap shows the distribution of stresses according to the color scale, while the maximum and minimum values for stresses are also denoted.

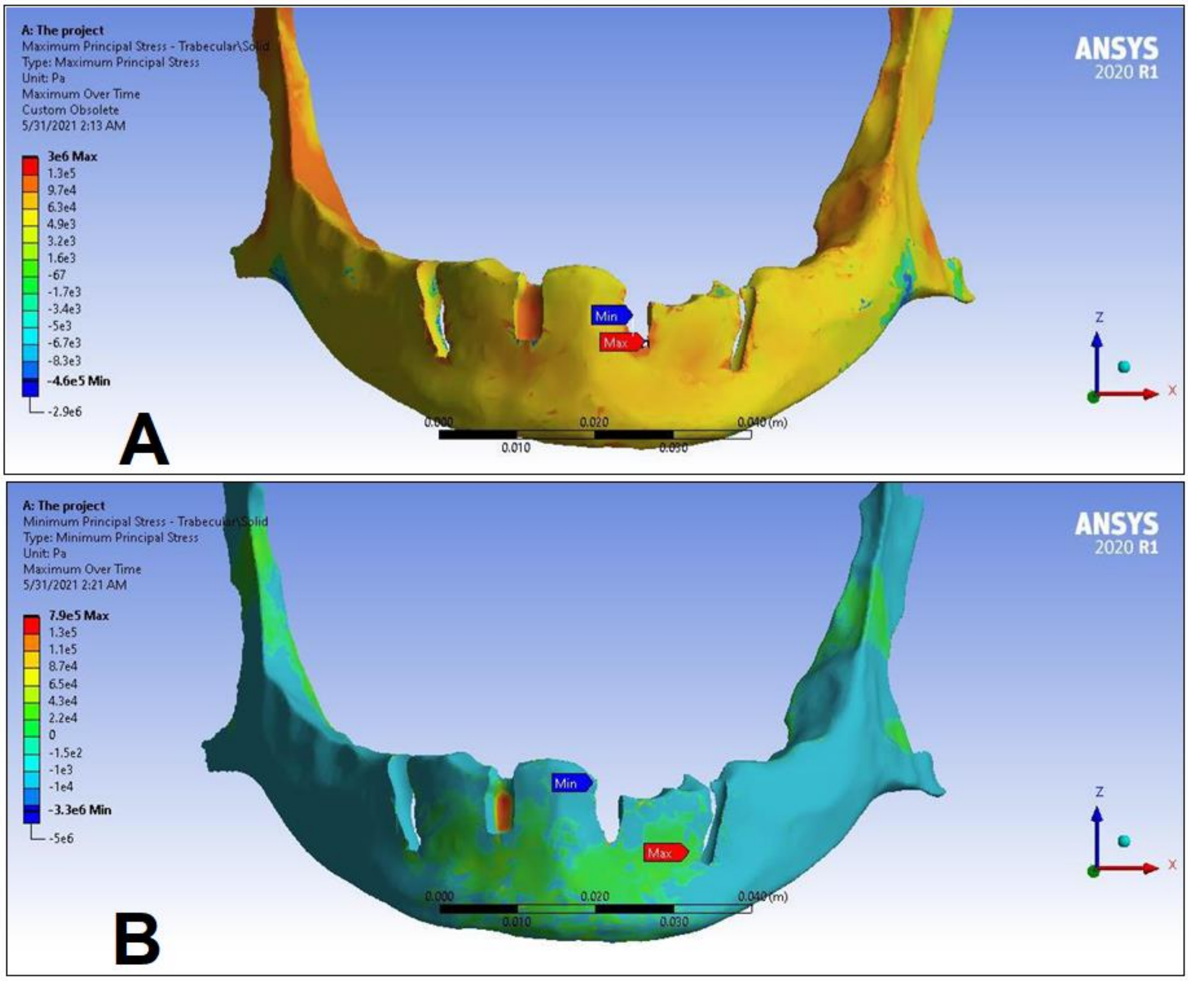

Supplementary figure S3. Maximum (P_{max}, A) and minimum (P_{min}, B) principal stress distributions in the trabecular bone segment of the mandible for the S1 OC2 case. The heatmap shows the distribution of stresses according to the color scale, while the maximum and minimum values for stresses are also denoted.

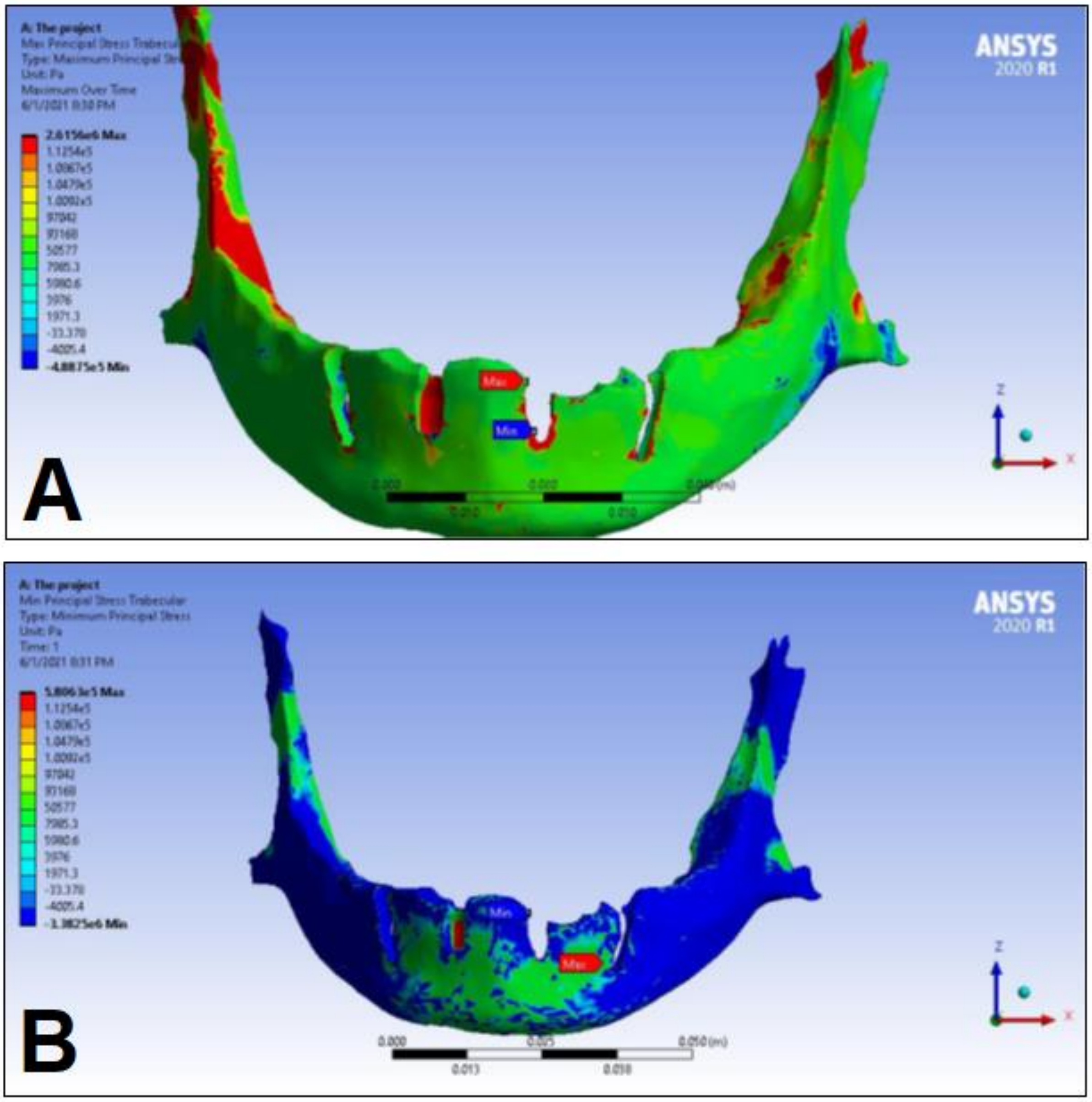

Supplementary figure S4. Maximum (P_{max}, A) and minimum (P_{min}, B) principal stress distributions in the trabecular bone segment of the mandible for the S2 OC2 case. The heatmap shows the distribution of stresses according to the color scale, while the maximum and minimum values for stresses are also denoted.

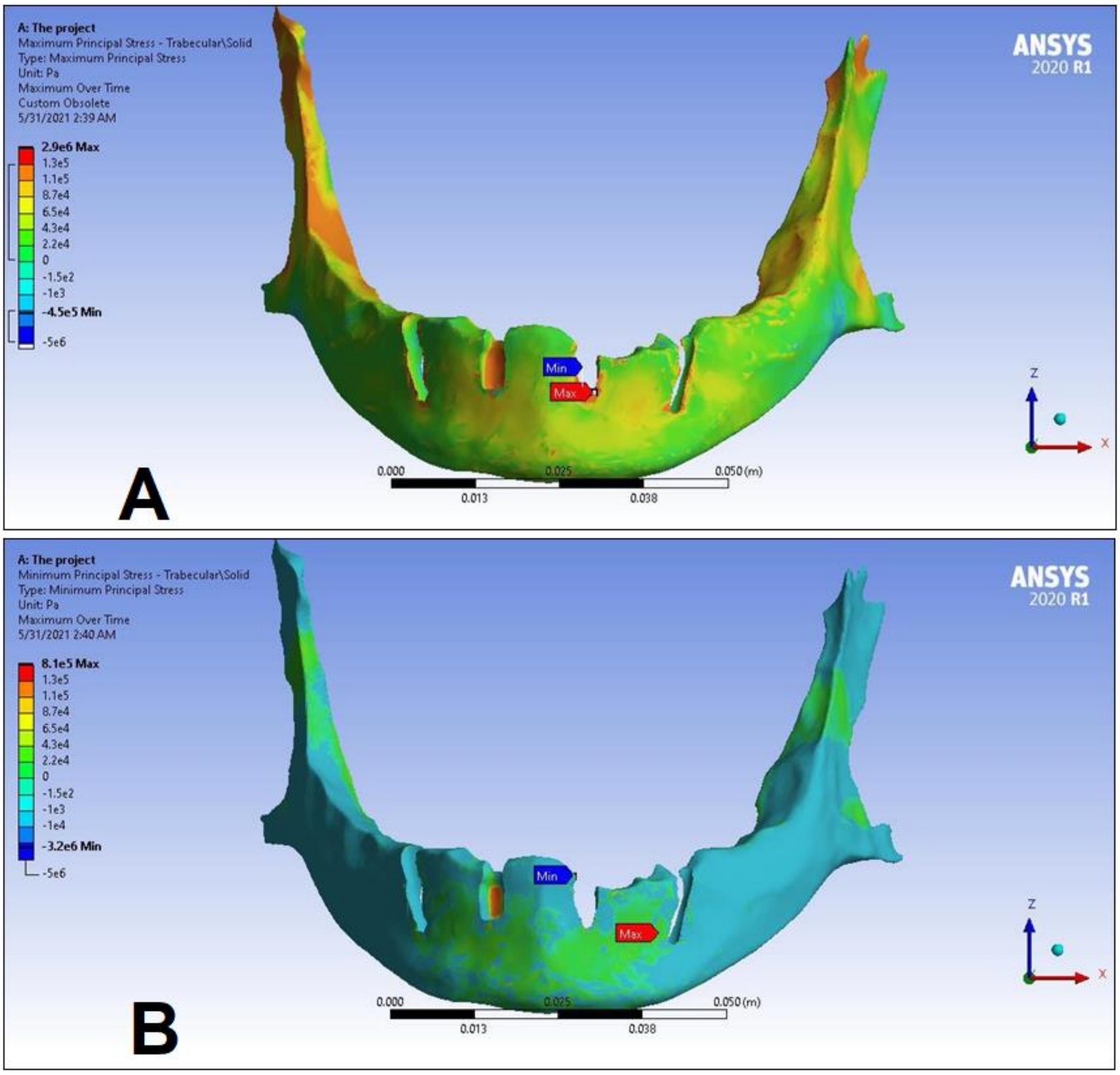

Supplementary figure S5. Maximum (P_{max}, A) and minimum (P_{min}, B) principal stress distributions in the trabecular bone segment of the mandible for the S1 OC3 case. The heatmap shows the distribution of stresses according to the color scale, while the maximum and minimum values for stresses are also denoted.

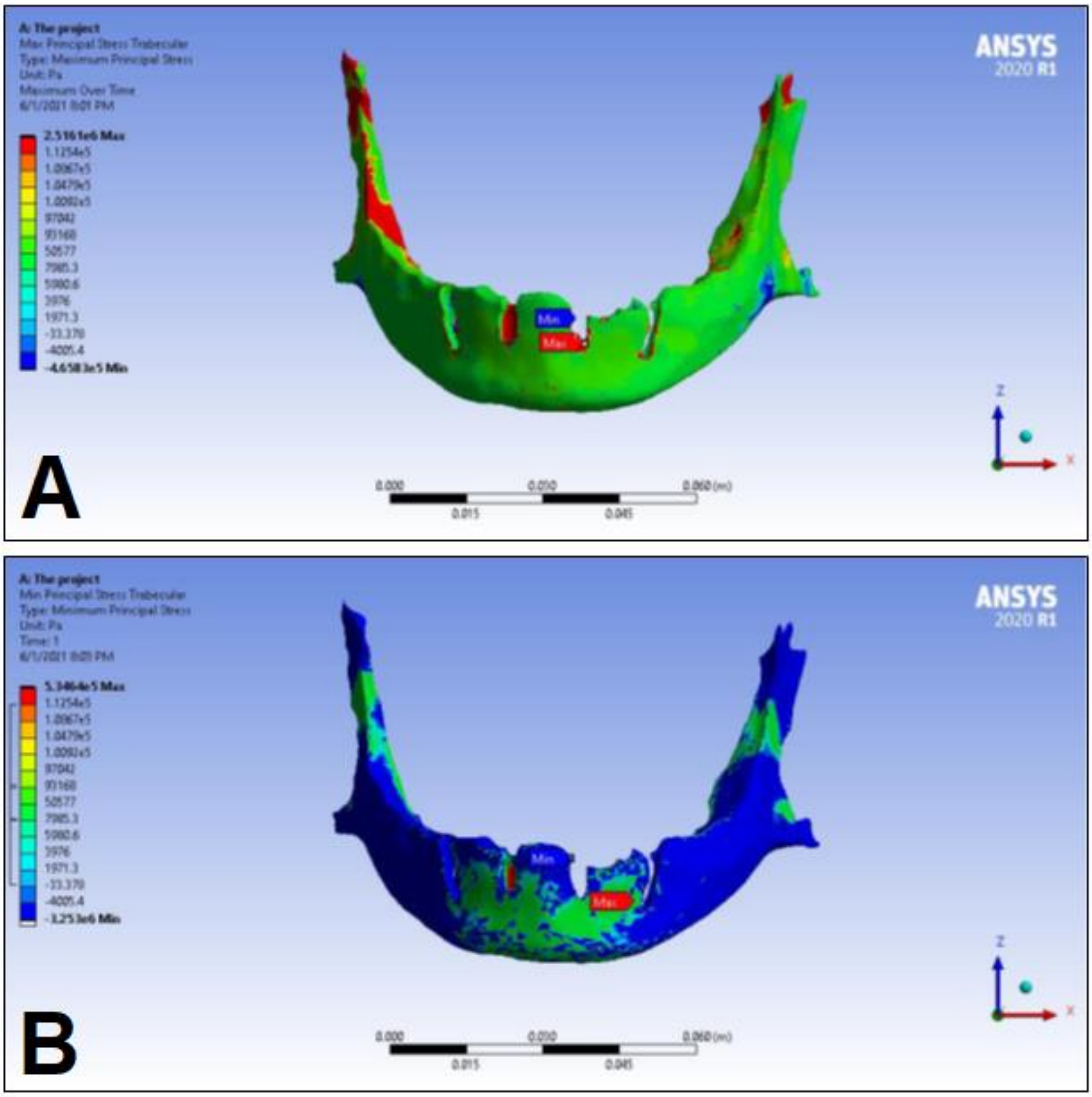

Supplementary figure S6. Maximum (P_{max}, A) and minimum (P_{min}, B) principal stress distributions in the trabecular bone segment of the mandible for the S2 OC3 case. The heatmap shows the distribution of stresses according to the color scale, while the maximum and minimum values for stresses are also denoted.

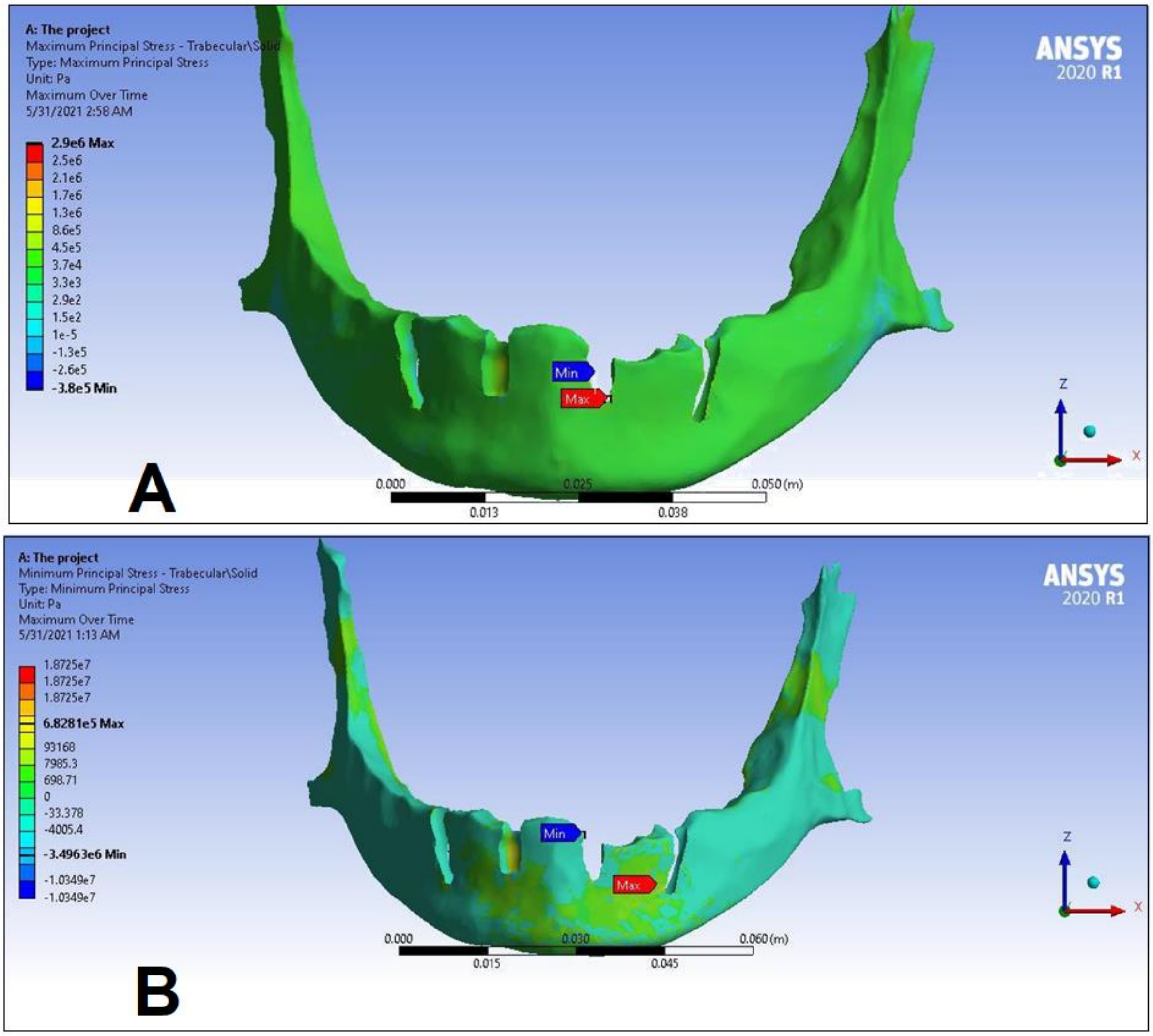

Supplementary figure S7. Maximum (P_{max}, A) and minimum (P_{min}, B) principal stress distributions in the trabecular bone segment of the mandible for the S1 OC4 case. The heatmap shows the distribution of stresses according to the color scale, while the maximum and minimum values for stresses are also denoted.

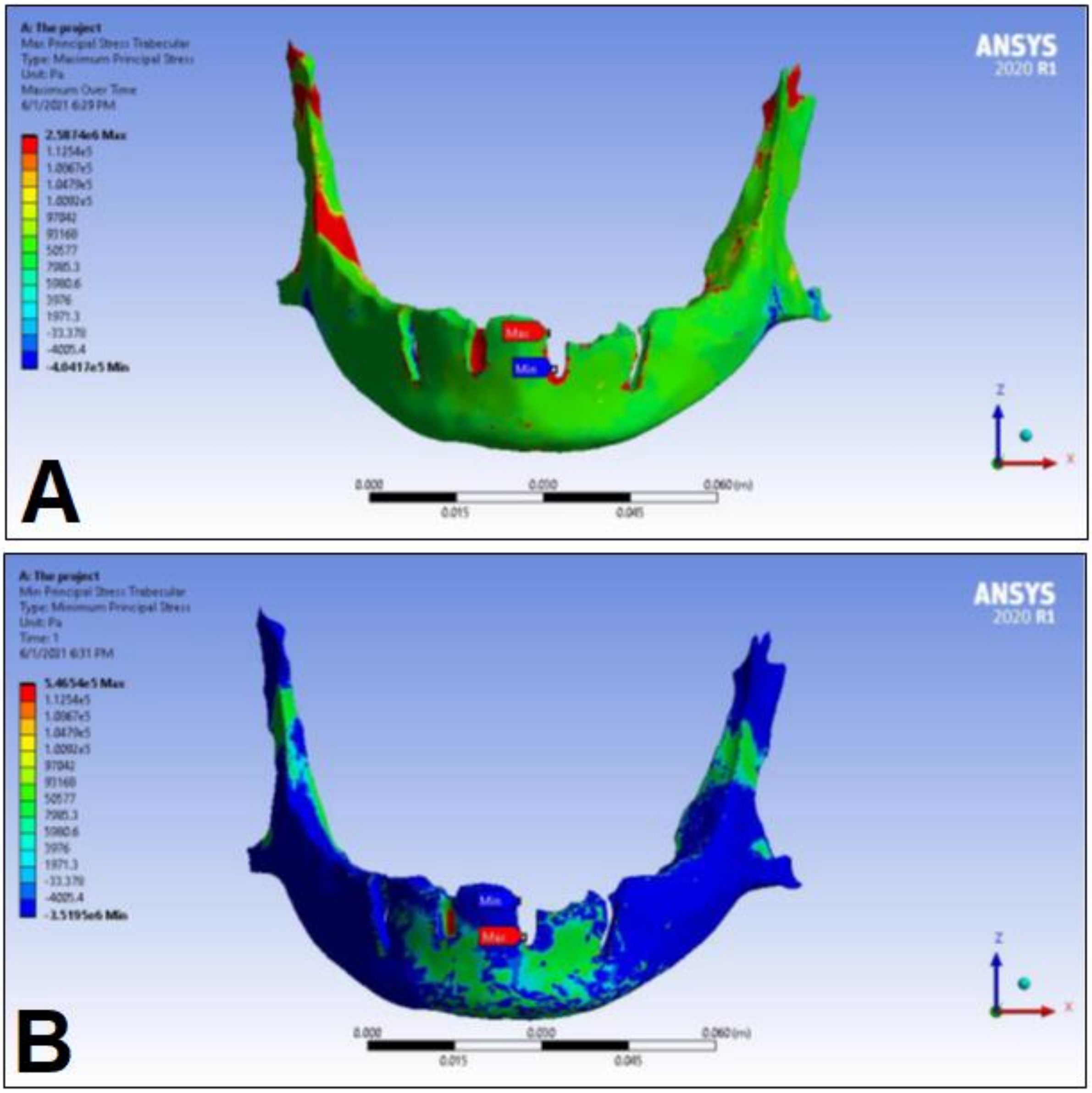

Supplementary figure S8. Maximum (P_{max}, A) and minimum (P_{min}, B) principal stress distributions in the trabecular bone segment of the mandible for the S2 OC4 case. The heatmap shows the distribution of stresses according to the color scale, while the maximum and minimum values for stresses are also denoted.
